# Sleep quality, valence, energetic arousal, and calmness as predictors of device-based measured physical activity during a three-week mHealth intervention: An ecological momentary assessment study within the SMART*FAMILY* trial

**DOI:** 10.1007/s12662-022-00809-y

**Published:** 2022-04-14

**Authors:** Janis Fiedler, Caroline Seiferth, Tobias Eckert, Alexander Woll, Kathrin Wunsch

**Affiliations:** 1 Institute of Sport and Sport Science, Karlsruhe Institute of Technology, 76131 Karlsruhe, Germany; 2 Department of Clinical Psychology and Psychotherapy, University of Bamberg, 96047 Bamberg, Germany; 3 Heidelberg Institute of Global Health (HIGH), Heidelberg University, 69117 Heidelberg, Germany

**Keywords:** Accelerometry, Mobile health, Ambulatory assessment, Affective states, Health behavior

## Abstract

Physical inactivity is known to be a risk factor for several noncommunicable diseases and has a high prevalence in today’s society. Therefore, it is crucial to understand the psychological factors associated with physical activity (PA). Recent developments in the field of ambulatory assessment and technological advances are promising to enhance our understanding of this relationship by analyzing longitudinal data within- and between-persons. These analyses can reveal important factors to design behavior change interventions to enhance PA. Therefore, this study used an ecological momentary assessment during the 3‑week intervention period in the SMART*FAMILY*2.0 trial and aimed to investigate whether valence, calmness, energetic arousal, and sleep quality predict daily steps and moderate to vigorous PA. Overall, 49 adults (35–60 years) and 40 children (5–19 years) were included in this analysis and self-rated their mental state within our smartphone application while also wearing a hip-worn accelerometer for 21 consecutive days (996 days included) during the intervention period. Multilevel analyses were conducted to predict daily PA while considering covariables (e.g., child/adult and non-wear time) both within- and between-persons. The results indicated that higher than average ratings of a person’s valence and energetic arousal on one day predicted increased PA while higher than average calmness predicted decreased PA at the same day within this person. Sleep quality and between-person effects of the affective states showed no clear associations to PA. Overall, these results showed that within-person associations of valence, calmness, and energetic arousal should be considered when designing PA interventions for both children and adults. The influence of sleep quality, as well as between-person effects, should be further explored by future studies.

**Supplementary Information:**

The online version of this article (10.1007/s12662-022-00809-y) contains supplementary material, which is available to all users.

## Introduction

Physical inactivity depicts one major risk factor for a variety of noncommunicable diseases (Kohl et al., 2012) while sufficient physical activity (PA) represents an effective primary prevention strategy for noncommunicable diseases throughout the lifespan (Beaglehole et al., 2011). However, only 32% of the worldwide population reach the PA recommendations of 150 min of moderate or 75 min of vigorous PA or an equivalent of both for adults (> 18 years) and an average of 60 min moderate to vigorous PA (MVPA) per day for children (5–17 years) (Bull et al., 2020; Hallal et al., 2012). Hence, effective interventions to reduce physical inactivity and to enhance PA are needed for adults and children to meet their respective guidelines. Today, mobile health (mHealth) interventions are promising tools for health behavior change due to preliminary results for effectiveness, 24/7 availability, extensive coverage, and their assumed cost-effectiveness (Vandelanotte et al., 2016). Important key facets for effective mHealth interventions are hereby the theoretical foundation, the use of behavior change techniques, interventions’ embeddedness in a social context, and individual tailoring (Fiedler, Eckert, Wunsch, & Woll, 2020). Besides these contextual and cognitive factors, there is a further need to investigate affect-related determinants in individuals assigned to a mHealth intervention targeting PA to identify reasons for uptake, or barriers, of subsequent PA (Dunton, 2017).

Ecological Momentary Assessment (EMA) provides an opportunity to not only deliver interventional content but also to gather real-time within- and between-person longitudinal data throughout the intervention period (Trull & Ebner-Priemer, 2013). This allows the detection of dynamic associations between determinants of subsequent PA on an individual level, which can be considered in personalized behavior change interventions (Conroy, Lagoa, Hekler, & Rivera, 2020). Of particular interest are hereby dimensions of affect that are assumed to be linked to an improved health behavior (Trull & Ebner-Priemer, 2013). There is much contradiction and overlap in the conceptualization of affect, mood, and emotion (for a review, see Ekkekakis, 2013). James Russel (2003) proposed a framework that establishes interrelationships between these concepts and defined core affect as a “neurophysiological state consciously accessible as a simplest raw (nonreflective) feeling evident in moods and emotions” (Russell, 2003, p. 148). Building on this, different models and dimensions of core affect have been postulated in recent years. According to the three-dimensional model, core affect includes at least three basic intercorrelated affective dimensions that map the complexity of affective states in daily life: valence (pleasure–displeasure), energetic arousal (wakefulness–tiredness), and calmness (relaxation–tension) (Schimmack & Grob, 2000).

Extensive research has been conducted in the past years investigating the relationship between PA and core affect in adult populations (Forster et al., 2021; Liao, Shonkoff, & Dunton, 2015). Previous research indicates that valence (Carels, Coit, Young, & Berg, 2007; Emerson, Dunsiger, & Williams, 2018; Kanning & Schoebi, 2016; Schwerdtfeger, Eberhardt, Chmitorz, & Schaller, 2010) and energetic arousal (Liao, Chou, Huh, Leventhal, & Dunton, 2017; Niermann, Herrmann, von Haaren, van Kann, & Woll, 2016; Schwerdtfeger et al., 2010) are positively associated with subsequent PA, while calmness is negatively associated with PA (Kanning & Schoebi, 2016; Reichert et al., 2016). Although the results seem to be coherent on the affective dimensions, a direct comparison is difficult because the studies analyzed different temporal aspects of subsequent activity (i.e., 24 h, 15 min) and different types of movement (i.e., free-living PA vs. structured exercises) (Forster et al., 2021). For example, Carels et al. (2007) and Emerson et al. (2018) investigated the relationship between affect and PA within a single day and the results indicate that higher ratings of valence in the morning were associated with increased PA over the day. Here, both studies assessed PA by self-report, which may not represent changes within an individual in detail (Reichert et al., 2020) and often differs from device-based measured PA (Fiedler, Eckert, Burchartz, Woll, & Wunsch, 2021). Comparable results for the relation of PA and energetic arousal alone were also found in children between 9 and 13 years (Dunton et al., 2014) and for all three affective states in children between 12 and 17 years (Koch et al., 2018). Despite these findings, it is important to note that the dynamic relationship between affective states and PA has been studied much less in children and that the existing results are heterogeneous (Bourke, Hilland, & Craike, 2021). In addition, parameters of sleep (i.e., perceived sleep quality, duration, efficacy) are further important determinants of health-related behavior that are assumed to be linked with PA (Wang & Boros, 2021). However, a recent meta-analysis including adult samples revealed no direct relationship between sleep on subsequent PA (Atoui et al., 2021), while a longer sleep duration was associated with improved eating behavior and higher levels of PA in children (Khan, Chu, Kirk, & Veugelers, 2015).

As stated above, the dimensions of core affect and perceived sleep quality can influence PA behavior in both adults and children. Therefore, it is important to investigate these covariates during a theory-based intervention in which key facets of behavior change are implemented. This can help to assess the possible impact of affective states and sleep quality on the main outcome (PA) of the intervention. Here, existing studies have mainly evaluated EMA-measured constructs as time-lagged predictors immediately before PA uptake to investigate their momentary effect (Liao et al., 2015). However, in the intervention context day-level peculiarity might also be of interest, as intervention studies usually include time intervals of several days to weeks, and the question if EMA-derived variables have an impact on this time scale is important for designing such interventions. Another important point is to take the PA outcome into account. Here, a study by Reichert et al. (2017) found differences in the relationship of PA to affective states for exercise and nonexercise PA which suggests that there is no uniform relationship between PA and affect. Knowledge of the mechanisms and barriers related to PA uptake during a longer measurement period will also help to anticipate mental health- and sleep quality-related barriers causing physical inactivity which can then be considered for the development of future mHealth interventions (Dunton, 2017).

Hence, the present study aimed to investigate several potential mental health-related covariates of PA including valence, energetic arousal, and calmness as well as perceived sleep quality on a daily level during 3 weeks to predict same-day PA measured by (1) steps, and (2) MVPA, among children and adults during a PA intervention period. These two PA measures were used to account for possible differences in the relationship between an intensity independent (steps) and intensity-related (MVPA) PA measure, and to project two different types of PA guidelines: the step-related guideline of reaching between 7000 and 10,000 steps per day (e.g., Paluch et al., 2021), which is followed by most people using fitness trackers or smartwatches as a daily goal, and the intensity-related guideline provided by the World Health Organization (Bull et al., 2020).

Following previous findings on the topic, it is hypothesized that on days where participants report higher than usual valence and energetic arousal, they have greater device-based measured step count and MVPA on the same day while on days where participants report higher than usual calmness, they have lower device-based measured step count and MVPA on the same day (within-persons). Between-person effects of valence, energetic arousal, and calmness on steps and MVPA (e.g., participants who report higher valence on average have higher/lower average device-based measured step count compared to persons who report lower valence on average), and the relationship between sleep quality and PA on a within- and between-person level will be explored.

## Methods

### Participants and procedure

Data for the current study were based on the SMART*FAMILY*2.0 trial. The detailed study protocol of the SMART*FAMILY*2.0 study has been previously published (Wunsch et al., 2020). Full ethical approval and written informed consent of all participants, children, and legal guardians were obtained (The International Registered Report Identifier [IRRID] for the SMART*FAMILY* study is RR1-10.2196/20534.). The trial was conducted in accordance with the Declaration of Helsinki.

Participants (families) were recruited in schools, school holiday programs, music schools, and sports clubs via personal communication, newspapers, and email distribution lists of the Karlsruhe Institute of Technology. Only families including at least one parent and at least one child who was 10 years of age or older and who were living together in a common household were eligible for the study. In addition, all siblings were invited to take part in the study if the parent(s) vouched for their ability to participate (Wunsch et al., 2020). All participants have been cluster-randomized into an intervention group and a control group. The protocol for both groups included a baseline measurement of 1 week, followed by a 3-week intervention/waiting period and 1‑week post measurement. The original study aimed to enhance PA and healthy eating with the digital intervention. For this study, only data of the 3‑week intervention period in the intervention group (*n* = 98, 52% adults) has been included. During this intervention period, participants used the SMART*FAMILY*2.0 app on provided smartphones and wore an accelerometer. To increase participants’ health literacy, information about the benefits of PA and healthy eating was provided in the app. In addition, participants autonomously set activity- and diet-related weekly goals, received feedback on goal achievement, and received a just-in-time adaptive intervention (i.e., a push notification when the participant was inactive during the wake time for at least 60 min (neither < 2 sensor values at > 2 MET nor 100 steps registered on the accelerometer); for an overview of just-in-time adaptive interventions see Wunsch, Eckert, Fiedler, & Woll, 2022). EMA concerning sleep quality was sent once in the morning (i.e., the first action of the participant on the app each day), and EMA concerning affect after a period of inactivity (following the just-in-time adaptive intervention) and, if no trigger occurred for several hours, in the evening.

### Measurements

#### Accelerometry

PA (i.e., steps and MVPA per day) was continuously recorded by 3‑axial accelerometers (Move 3/Move 4, Movisens GmbH, Karlsruhe, Germany). The small-scale (62.3 mm × 38.6 mm × 11.5 mm) and light-weight accelerometers were worn at the right hip and were attached by a clip or on a belt. Raw data were sampled at an input frequency of 64 Hz and afterward summarized in 60 s epochs. Analyzed raw data were processed by algorithms into steps, time spent during MVPA minutes per day [> 3 metabolic equivalents (MET)], inactive time [1–1.5 MET], and non-wear time for this study. The accelerometers have been shown to accurately detect step counts (Anastasopoulou, Härtel, & Hey, 2013) and to validly estimate energy expenditure (Anastasopoulou et al., 2014).

Participants were instructed to wear the accelerometer during wake time for the whole intervention period of 3 weeks, with each measurement period starting on a Monday. Participants were told to remove the sensors during showering, swimming, or during contact sports. In this case, the participants were instructed to manually record the duration and intensity of the exercise in the SMART*FAMILY*2.0 app (not included in our study).

#### Ecological momentary assessment

Several EMAs were assessed within the study. Participants were instructed to use the app throughout the day and only mute it during, for example, meetings or school. With the first action on the app in the morning, every participant rated the perceived sleep quality once a day on a 7-point Likert scale (“How would you rate your sleep quality during the previous night?” 0 = very bad, 6 = very good, adapted from Snyder, Cai, DeMuro, Morrison, and Ball (2018)). Wilhelm & Schoebi, 2007 previously showed that two bipolar items each provide sensitive and reliable measurements of the three-dimensional model of core affect (Schimmack & Grob, 2000). In this study, only one of those two bipolar items was used asking for affective valence (“How is your current mood?” rated by emojis from 0 = very bad, to 4 = very good), energetic arousal (“Are you feeling awake or tired?” 0 = very tired, 6 = very awake), and calmness (“Are you feeling relaxed/calm or stressed?” 0 = very stressed, 6 = very calm). The items were used based on Bachmann et al. (2015) to keep participant burden low. The use of single items can hereby be beneficial for research focused on a broader perspective of the relationship between affect and PA even though it limits conclusions about discrete affects (Emerson et al., 2018). The EMA concerning affective states was sent following an event-contingent scheme when participants were inactive during the last 60 min (neither > 2 sensor values at > 2 MET nor 100 steps), and when the participant finished their day in the app by pressing the “going to sleep button” (provided no trigger occurred during the past hour). The inactivity triggers were blocked when (1) the app was “asleep”, (2) during the night (10 pm to 7 am), (3) less than 50 of 60-minute values have been sent during the past hour by the sensor, and (4) if a participant reached a PA level of 60 min of MVPA on a certain day. As EMAs could be sent multiple times a day, daily averages were calculated for valence, energetic arousal, and calmness. Here, it needs to be noted that this study is a secondary data analysis of the intervention period in a free-living study, where participants were not instructed to answer a certain amount of EMA questionnaires. Therefore, the interaction with the app does not represent compliance as in other EMA studies but user engagement with the app (comparable to, for example, Edney et al., 2019).

### Statistical analysis

R (R Core Team, 2021) and RStudio (RStudio Team, 2021) were used for data preparation and analysis. The package ‘ggplot2’ was used for visualizations (Hadley Wickham, 2016). Due to the hierarchal structure of the data multilevel models were calculated using the package ‘nlme’ (Jose Pinheiro, Bates, DebRoy, Sarkar, & R Core Team, 2021) with days of the intervention (level 1) nested in participants (level 2) to identify the within- and between-person effects concerning the research question. The result tables of the regression analyses were generated using the package ‘sjPlot’ (Daniel Lüdecke, 2021). Here, two final models were calculated, one with each PA parameter (steps and MVPA per day) as outcome variables. Intraclass correlation coefficients (ICCs) of the null model indicated that 40% and 54% variances for each of the steps and MVPA, respectively, were due to within-person differences. Therefore, the influence of the hierarchal data structure on the outcome variables was confirmed and a multilevel approach was used. ICCs for the predictor variables indicated that between 60% and 71% of variance was explained by within-person differences and the variables were therefore disaggregated into within- and between-person variables. Assumptions were checked using the visualization of the ‘performance’ package (Daniel, Mattan, Indrajeet, Philip, & Dominique, 2021). If the assumptions seemed to be violated, a robust model was fitted using the package ‘robustlmm’ (Manuel Koller, 2016) and compared to the nonrobust version. Only the nonrobust model was reported as no noticeable difference emerged between both versions of the models. The need of controlling for autocorrelation was also checked which improved the model and was therefore included in all models. A hierarchical approach was used for the inclusion of the control variables and the model fit was assessed with −2 restricted log-likelihood and the Akaike information criterion (AIC). A sensitivity test was also performed where participants with less than six measurements (*n* = 24, 27%) were excluded from the analyses which yielded comparing β with similar significances. Therefore, the models including all 89 participants with valid measurements were used.

The predictors sleep quality, valence, energetic arousal, and calmness, and the control variable non-wear time were included at level 1 and centered at the person-mean to estimate within-person effects (Hoffman & Stawski, 2009). In addition, the control variable weekday or weekend (i.e., weekday = 0, weekend = 1) was included in the models at level 1. Time (i.e., day of the study 0–20) was added as a within-person control variable at level 1 but showed no significant effect and was not included in the final models. The mean scores per person for each level 1 predictor were added as level 2 predictors to unravel the between-person from the within-person results (Hoffman & Stawski, 2009). Adult/child (i.e., adult = 0, children = 1) was added as a between-person control variable at level 2. Sex (i.e., female = 0, male = 1) was only added for MVPA as a between-person control variable at level 2 as it did not improve the model for steps. Random slopes were computed for all level 1 predictors which did not improve the models and were therefore excluded in the final models. Random intercepts were used for both models and the level for significance was set a priori to α < 0.05.

The following equations of the final models (with the only difference that sex was excluded for the steps model) were used:

Level 1 equation:

$$Y_{ij}=\beta _{0j}+\beta _{1j}\times \left(\textit{sleep}\,\textit{quality}\right)_{ij}+\beta _{2j}\times \left(\textit{valence}\right)_{ij}+\beta _{3j}\times \left(\textit{calmness}\right)_{ij}+\beta _{4j}\times \left(\textit{energetic}\,\textit{arousal}\right)_{ij}+\beta _{5j}\times \left(non-weartime\right)_{ij}+\beta _{6j}\times \left(wewd\right)_{ij}+r_{ij}$$

Level 2 equation:

$$\beta _{0j}=\gamma _{00}+\gamma _{01}\times \left(mean\,\textit{sleep}\,\textit{quality}\right)_{j}+\gamma _{02}\times \left(mean\,\textit{valence}\right)_{j}+\gamma _{03}\times \left(mean\,\textit{calmness}\right)_{j}+\gamma _{04}\times \left(mean\,\textit{energetic}\,\textit{arousal}\right)_{j}+\gamma _{05}\left(\textit{adult}/\textit{child}\right)+\gamma _{06}\,\left(sex\right)+u_{0j}$$

where $$\beta _{1j}=\gamma _{10}$$, $$\beta _{2j}=\gamma _{20}$$, $$\beta _{3j}=\gamma _{30}$$, $$\beta _{4j}=\gamma _{40}$$, $$\beta _{5j}=\gamma _{50}$$, and $$\beta _{6j}=\gamma _{60}$$.

## Results

### Data availability and participant characteristics

Overall, 98 participants received a total of 2058 sleep quality EMAs over the 21-day collection period. The average number of sleep quality ratings completed by each participant was 17.39 out of 21, equating to 82.85% complete data (represents daily app use). On 1332 of 2058 days, the participants additionally answered at least one EMA assessing valence, energetic arousal, and calmness (averaged from 2579 triggers). This implied that daily mean values for each affective state and each participant could be calculated for 64.72% of the days. Days (*n* = 775/2058) with missing values (2531 data points) in either sleep quality or affect ratings were excluded from the final analyses.

In addition, 656/2058 days indicated either greater non-wear time than 960 min (618 data points) or that more than 1200 min were classified as an energy-expenditure range of 1.0 to 1.5 METs (34 data points) or that zero step counts were recorded (285 data points). Those days were also excluded from the analysis (some of which overlapped with the excluded days for sleep quality and/or affect).

The exclusion of days due to missing and invalid data points resulted in a final analytic sample of 49 adults (35–60 years) and 40 children (5–19 years) and a total of 996 days (adults = 661; children = 335), yielding an average of 11.19 valid measurement occasions per participant (affect triggers were summarized from 1–9 measurements per day). Participant characteristics for the final sample are shown in Table 1. A daily overview of all outcomes and predictors divided by adults and children is shown in supplement figure 1. Here, the variability of daily data and individual patterns of the variables over time for each participant can be inspected in supplement figures 1–7. The mean body mass index (BMI) was 25.38 (standard deviation [SD] = 3.91) kg/m^2^ in the adult and 17.62 (SD = 2.92) kg/m^2^ in the children population.

**Table 1:** Descriptive data of all participants included in the analyses. Displayed are the means and standard deviations (SD) during 3 weeks for the parameters age, steps, moderate to vigorous physical activity (MVPA), non-wear time (nwt), self-rated sleep quality (sleep), self-rated valence, self-rated energetic arousal (energetic), and self-rated calmness

Population	Adult		Child	
Sex	Female (*n* = 24)	Male (*n* = 25)	Female (*n* = 23)	Male (*n* = 17)
	Mean (SD)	Mean (SD)	Mean (SD)	Mean (SD)
Age (years)	44.8 (5.4)	46.6 (5.1)	11.2 (2.8)	11.9 (3.9)
Steps (count/day)	7880 (2600)	6700 (2540)	8280 (3230)	9890 (2940)
MVPA (min/day)	56.0 (25.7)	59.3 (29.4)	54.7 (45.2)	117 (36.3)
nwt (min/day)	641 (84.5)	630 (98.1)	703 (98.9)	744 (96.3)
Sleep (0–6)	4.25 (0.92)	3.82 (0.82)	4.14 (1.50)	4.51 (0.94)
Valence (0–4)	2.82 (0.38)	2.89 (0.45)	3.09 (0.47)	3.29 (0.61)
Energetic (0–6)	3.89 (0.67)	4.07 (0.74)	4.13 (1.21)	4.82 (0.93)
Calmness (0–6)	2.71 (0.844)	2.98 (0.845)	2.73 (1.13)	3.04 (1.22)

### Effects of sleep quality and affective states on daily step count

#### Within-person effects (level 1)

Results indicate no significant within-person effects between sleep quality ratings and daily step count (Table 2). As hypothesized, the daily average affective states rated by a person were associated with the number of device-based measured steps per day. In detail, a higher than average rating of a person’s valence on one day significantly predicted a higher step count on the same day within this person (β = 0.06,* p* = 0.024). In practice, a 1-point increase in valence above the person-mean (original scale 0–4) was related to an average increase of 489.63 more steps on the same day. Furthermore, higher than average values of a person’s energetic arousal ratings were related to an increase in that person’s device-based step count on the same day (β = 0.07, *p* = 0.014). As expected, days with higher than average ratings of calmness within a person were associated with significantly lower device-based measured step count on the same day (β = −0.07, *p* = 0.007). The daily non-wear time showed a significant effect on steps. This means that on a day when a person wore the accelerometer 1 min less than their person-based average, the accelerometer recorded 4.09 fewer steps (*p* < 0.001) for the same person. In addition, the number of recorded steps of a person was significantly higher on weekend days than on weekdays (β = 0.09, *p* = 0.001).

**Table 2:** Multilevel model analysis for the influences of sleep quality and affective states on daily step count. Displayed are the within-person results (wp) of the person-mean centered variables self-rated sleep quality (sleep; original range 0–6), self-rated valence (valence; original range 0–4), self-rated energetic arousal (energetic; original range 0–6), and self-rated calmness (calmness; original range 0–6) and the within-person, person-mean centered control variable non-wear time (nwt) and the variable weekend/weekday (wewd; weekday = 0, weekend =1). In addition, the between-person results (bp) of the affective states and sleep quality, and the influence of adult/child (adult = 0, children = 1) on steps are shown. All results are displayed using the raw Beta (B), the standardized Beta (β), 95% confidence intervals (CI), standardized (std.) 95% CI, and the level of significance (*p*) where bold numbers indicate α < 0.05. The within-person variance (σ^2^), the between-person variance (τ_00_ id), the intraclass correlation coefficient (ICC), the number of participants (N _id_), and the number of observations are also displayed

Steps					
Predictors	B	β	CI	Std. CI	*p*
(Intercept)	6157.69	−0.02	2263.20–10,052.18	−0.16–0.11	**0.002**
Wp_sleep	130.57	0.03	−52.73 to 313.87	−0.01–0.08	0.165
Wp_valence	489.63	0.06	66.15–913.10	0.01–0.12	**0.024**
Wp_calmness	−244.11	−0.07	−418.90 to −69.32	−0.11–−0.02	**0.007**
Wp_energetic	339.23	0.07	71.45–607.01	0.01–0.12	**0.014**
Wp_nwt	−4.09	−0.11	−5.90 to −2.28	−0.16–−0.06	**<** **0.001**
Wewd	848.32	0.09	340.50–1356.15	0.03–0.14	**0.001**
Bp_sleep	49.77	0.01	−587.52 to 687.05	−0.12–0.15	0.878
Bp_valence	843.16	0.09	−977.53 to 2663.85	−0.10–0.28	0.363
Bp_calmness	−778.42	−0.16	−1442.62 to −114.21	−0.29–−0.02	**0.023**
Bp_energetic	147.51	0.03	−818.79 to1113.81	−0.15–0.20	0.764
Bp_adult/child	1452.05	0.16	220.21–2683.90	0.02–0.30	**0.022**
**Random effects**					
σ^2^	10,220,975.78				
τ_00 id_	5,595,387.95				
ICC	0.35				
N _id_	89				
Observations	996				

#### Between-person effects (level 2)

Results showed no significant between-person effects between sleep quality, valence or energetic arousal ratings, and device-based measured step count. However, individuals with higher average calmness ratings had significantly fewer daily steps recorded when compared to individuals with lower averages (β = −0.16, *p* = 0.023). Furthermore, significant differences between children and adults in the average number of steps per day (β = 0.16, *p* = 0.022) were found insofar as the accelerometers recorded 1452.05 more steps a day on average in children than in adults. Overall, the ICC showed that 35% of the variance in the model was due to between-person and 65% due to within-person variance.

### Effects of sleep quality and affective states on daily MVPA

#### Within-person effects (level 1)

As shown in Table 3, neither perceived sleep quality (*p* = 0.434) nor mean energetic arousal (*p* = 0.070) ratings of one day were associated with MVPA during the same day within a person. As hypothesized, the daily average valence ratings per day significantly predicted higher MVPA (β = 0.07, *p* = 0.006). In practice, a 1-point increase in valence above the person-mean (original scale 0–4) was related to an average increase of 6.55 more minutes of MVPA on the same day. Furthermore, days with higher than average ratings of calmness within a person were associated with significantly lower device-based measured time spent in MVPA (β = −0.04, *p* = 0.035). In addition, the analyses revealed that non-wear time significantly predicted lower daily recorded MVPA (β = −0.07, *p* = 0.001) and higher MVPA was recorded during weekend days compared to weekdays (β = 0.06, *p* = 0.006).

**Table 3:** Multilevel model analysis for the influences of sleep quality and affective states on daily moderate to vigorous physical activity (MVPA). Displayed are the within-person results (wp) of the person-mean centered variables self-rated sleep quality (sleep; original range 0–6), self-rated valence (valence; original range 0–4), self-rated energetic arousal (energetic; original range 0–6), and self-rated calmness (calmness; original range 0–6) and the within-person, person-mean centered control variable non-wear time (nwt), and the variable weekend/weekday (wewd; weekday = 0, weekend =1). In addition, the between-person results (bp) of the affective states and sleep quality, and the influence of adult/child (adult = 0, children = 1) and sex (0 = female, 1 = male) on MVPA are shown. All results are displayed using the raw Beta (B), the standardized Beta (β), 95% confidence intervals (CI), standardized (std.) 95% CI, and the level of significance (*p*) where bold numbers indicate α < 0.05. The within-person variance (σ^2^), the between-person variance (τ_00_ id), the intraclass correlation coefficient (ICC), the number of participants (N _id_), and the number of observations are also displayed

MVPA					
Predictors	B	β	CI	Std. CI	*p*
(Intercept)	66.72	−0.03	14.70–118.74	−0.18–0.12	**0.013**
Wp_sleep	0.81	0.02	−1.20–2.81	−0.03–0.06	0.434
Wp_valence	6.55	0.07	1.91–11.19	0.02–0.12	**0.006**
Wp_calmness	−2.07	−0.04	−3.98–−0.15	−0.09–−0.00	**0.035**
Wp_energetic	2.72	0.04	−0.21–5.65	−0.00–0.09	0.070
Wp_nwt	−0.03	−0.07	−0.05–−0.01	−0.12–−0.03	**0.001**
Wewd	7.89	0.06	2.29–13.49	0.02–0.11	**0.006**
Bp_sleep	0.65	0.01	−7.94–9.24	−0.13–0.16	0.881
Bp_valence	−5.85	−0.05	−30.29–18.59	−0.25–0.15	0.637
Bp_calmness	−7.41	−0.12	−16.37–1.55	−0.26–0.03	0.106
Bp_energetic	2.42	0.04	−10.70–15.54	−0.16–0.23	0.717
Bp_adult/child	28.40	0.26	11.69–45.12	0.11–0.41	**0.001**
Bp_sex	29.84	0.28	13.64–46.04	0.13–0.44	**<** **0.001**
**Random effects**					
σ^2^	1249.24				
τ_00 id_	1101.01				
ICC	0.47				
N _id_	89				
Observations	996				

#### Between-person effects (level 2)

Differences in person-mean ratings of sleep quality and affective states between-persons did not predict daily MVPA (Table 3). However, results for the control variables showed that children had recorded significantly more MVPA than adults (β = 0.26, *p* = 0.001). In addition, significant sex differences (*p* < 0.001) were found for daily MVPA where being male was associated with higher daily MVPA values. Overall, the ICC showed that 47% of the variance in the model was due to between-person and 53% due to within-person variance.

## Discussion

This study used EMA and accelerometry to evaluate the within-person effects of children’s and adults’ daily self-reported valence, energetic arousal, and calmness on device-based measured MVPA and step count of the same day. Furthermore, the between-person effects of these variables were explored along with the within- and between-person effects of perceived sleep quality on steps and MVPA. The results mainly confirmed the hypotheses that ratings above the person-mean for valence and energetic arousal increased PA, while calmness decreased PA on a within-person level. One exception was the relationship between energetic arousal and MVPA which was not significant (*p* = 0.07) but showed a standardized estimate in the hypothesized direction. For the exploration of sleep quality as well as between-person effects of the predictors, only calmness showed a significant prediction for steps indicating that participants who rated their calmness one point higher (scale 0–6) had recorded 778.42 fewer steps per day on average. In addition, the results of the included control variables showed significant effects. Here, being male (only for MVPA) showed the largest effect, followed by being a child, having increased accelerometer wear time, and the measurement being on a weekend day. The results of this study mainly confirm the findings of previous studies using time-lagged predictors, indicating that the relation between affect and PA is consistent through different age groups (Cushing et al., 2017; Dunton et al., 2014; Koch et al., 2018; Liao et al., 2017; Niermann et al., 2016; Reichert et al., 2016; Schwerdtfeger et al., 2010), that affect and PA results are related on a day level (Do, Wang, Courtney, & Dunton, 2021), and add that these findings also apply to intervention studies.

### Valence and physical activity

The use and definitions of mood and affect and their subitems have been used interchangeably in the literature (Liao et al., 2015; Niermann et al., 2016); therefore, the results of previous studies related to all mood dimensions and affective states are treated as equal in this paragraph to provide a broader picture even though they often assess different constructs (see Ekkekakis, 2013 for an overview). In our study, valence significantly predicted both steps and MVPA at the same day within-persons which is in accordance with some previous studies (Dunton et al., 2014; Koch et al., 2018; Liao et al., 2017; Niermann et al., 2016; Reichert et al., 2016; Schwerdtfeger et al., 2010), while another study did not find such a relation between affect and PA in adolescents (Cushing et al., 2017). This strengthens the view that valence should be considered in building up PA interventions and that valence is a promising target to tailor interventions to a persons’ needs in a randomized controlled trial (Conroy et al., 2020). If for example, an assessment of valence indicates a low rating in a person compared to the usual rating, the most promising intervention might not be to target PA directly, but to improve valence and by doing so increase the probability that the person will engage in PA throughout the day.

### Energetic arousal and physical activity

Energetic arousal predicted steps but not MVPA on the same day within-persons, while previous studies found an association for different PA measures as time-lagged predictors (Dunton et al., 2014; Koch et al., 2018; Liao et al., 2017; Reichert et al., 2016). Here, two studies, one in adults (Reichert et al., 2016) and one in adolescents (Koch et al., 2018), indicated that the relationship of energetic arousal to nonexercise activity is stable in a timeframe of up to 300 min. The relation of energetic arousal to MVPA, however, has only been found for shorter (i.e. ,30 min) timeframes in children (Dunton et al., 2014) and another study found no relationship between energy (measured as a single item on a 1–5 scale) and MVPA 15 or 30 min after the EMA assessment in adults (Liao et al., 2017). Therefore, the relation of energetic arousal and MVPA might follow a narrower time pattern, while the relationship to steps as a parameter without intensity indication seems to be more time stable. These results should be considered for PA interventions as the influence of energetic arousal on PA seems to depend on the PA outcome and/or intensity. In this case, including or targeting energetic arousal in an intervention seems to be most beneficial for nonexercise activity or overall PA outcomes like step count. Here, digital games could be used to enhance energetic arousal (Collins, Cox, Wilcock, & Sethu-Jones, 2019) which could then be followed by a prompt to engage in PA.

### Calmness and physical activity

Self-rated calmness predicted both reduced recorded step count and reduced recorded MVPA per day within-persons which confirms findings of previous studies using it as a time-lagged predictor (Koch et al., 2018; Reichert et al., 2016). This means that if a person rated their calmness higher on a certain day than their average calmness this person engaged in less PA on that day. Therefore, having calmness included in the context of positive affect or overall mood (e.g., in Liao et al., 2017) might influence the result of the construct. Here, future studies which aim to investigate the relation between affect and PA should measure calmness as a separate construct from positive affect. This indicates the need to explore the relation of different subcategories of affect and mood. Here, the timeframe in which self-rated calmness predicted less subsequent PA was measurable in up to 130 min in one study (Koch et al., 2018) and up to 140 min in another study (Reichert et al., 2016) which suggests fairly time stable results for this parameter. Therefore, calmness seems to be another affective state which has to be considered for PA interventions. It is however questionable to reduce calmness to influence PA because calmness is also an important factor for health (Huffziger et al., 2013). In this case, more context about general PA behavior of the person and covariates of health behavior along with a clearer picture of a dose–response relationship between covariates and PA behavior is needed to provide individualized recommendations for behavior change interventions.

### Affect and related parameters between-persons

The association between affective states, sleep quality, and PA on a between-person level has been explored by this study. None of the affective states were associated with the average recorded time spent in MVPA per day. Regarding daily step counts, significant between-person variations were found in individuals with higher calmness ratings. These results suggest that individuals who feel calmer on average had recorded fewer steps on average than individuals who feel more stressed. No between-person association between energetic arousal, valence, and daily step count was found. Therefore, the results indicate that days, where a person rated their daily valence or energetic arousal higher than their usual daily valence or energetic arousal (within-person), showed enhanced PA. However, there was no difference for persons who rated their valence or energetic arousal higher on average compared to persons who rated their valence or energetic arousal lower (between-person) concerning PA. Further research is needed to define the link between affective states and PA behavior on the between-person level under consideration of the within-person level to specify if the observed relation is due to individual differences of participants or changes over time within participants, or both (Dunton, 2017).

### Sleep quality and physical activity variation within- and between-persons

The exploratory within- and between-person analyses indicated no significant association between the subjectively assessed sleep quality and device-based measured daily step count or daily MVPA. These results fit the findings of previous experimental and cross-sectional studies, which showed inconsistent relations between various sleep characteristics (e.g., efficiency, duration) and PA outcomes in children and adults (Antczak et al., 2020; Semplonius & Willoughby, 2018). In another study, Eythorsdottir, Frederiksen, Larsen, Olsen, and Heitmann (2020) used device-based measured sleep and PA outcomes and found no significant association between children with different sleep duration and sleep efficiency and PA. One explanation for these findings could be the high heterogeneity in the measurements or outcomes (e.g., sleep quality, sleep duration, wake time, bedtime) used. Further studies need to be designed which examine the bidirectional and temporal aspects of the sleep–physical behavior associations. In addition, to understand the effects of sleep characteristics (e.g., duration, efficiency, latency) on PA in more detail, more long-term studies with objectively measured sleep parameters are needed (e.g., using heart rate variability (Stein & Pu, 2012)), which consider daily schedules and further motivational aspects of activity behavior. Furthermore, as sleep-related measures often differ between adults and children (e.g., earlier bedtime and longer periods of nocturnal sleep for children) even within parent–child dyads (Gau, Shur-Fen, & Merikangas, 2004), studies comparing the results of both groups separately would benefit the understanding of the association between sleep quality and PA.

### Control variables

The findings of this study suggest significant differences in both recorded steps and MVPA outcomes between children and adults. Here, children showed a higher mean step count per day and higher time in MVPA than adults. Future studies should also investigate whether the relationship between affective states, and sleep and PA differs due to, for example, developmental differences throughout the lifetime. In addition, the results also indicate that time spent in MVPA during one day differed between sexes, suggesting that men and boys spent more time per day in MVPA than women and girls. Descriptive data of this study shows that while boys move more than girls (also illustrated in recent research (McGovern, Drewson, Hope, & Konopack, 2020)), women had a higher step count than men which is overlaid by the difference between boys and girls and therefore only visible in sex- and child/adult-disaggregated data. These results suggest that age-related sex differences should be considered when designing, implementing, and evaluating PA interventions for children and adults (Schlund et al., 2021). Furthermore, our study found non-wear time and differences between weekdays and weekend days to influence both steps and MVPA. A higher non-wear time predicted less PA during the day and participants were more active on weekends compared to during weekdays. Those variables should always be considered when interpreting PA outcomes if data has been measured over several days even if data with a certain wear time (i.e., less than 8 h/day) were excluded.

### Limitations

There are some limitations of this study that have to be considered for the interpretation of the results. First, the data of this study were collected during the ongoing coronavirus disease 2019 (COVID-19) pandemic which might differ from results before the pandemic, as certain restrictions have probably influenced PA patterns (Stockwell et al., 2021) and people’s affective states (Panayiotou, Panteli, & Leonidou, 2021). However, data collection has only been conducted when schools were open to allow comparability of the data. Second, the study focusses on EMA in an intervention design that aimed at increasing PA of the participants (without directly targeting the predictor variables) and included also other factors like health literacy and goal setting. We accounted for this aspect by controlling for days in the study which showed no significant effect and by visualizing the variability within the measurements and the individual development in supplement figures 1–7. In addition, the EMA triggers for valence, energetic arousal, and calmness were sent after a period of inactivity was detected by the accelerometer in addition to a trigger in the evening when the participants finished the day in the applications (provided no trigger was sent during the previous hour). Therefore, the number of triggers sent per day (which responses were then averaged, range 1–9) varied between the days and persons. The large amount of missing data also needs to be addressed which limits the generalizability of the findings. However, as stated in the methods section, sensibility analyses yielded comparing results and multilevel approaches are fairly robust to missing data. Moreover, daily mean values were used in this study instead of time-lagged predictors which are important for the interpretation as PA and affect can have a bidirectional relationship (Liao et al., 2015). As it is not known whether the participants were active before, during, or after the assessments in this study, the multilevel modeling results concern the overall association of the measures on a certain day but are not related to the question of the time-related direction of the effect. Furthermore, it is unclear whether the daily mean values for valence, energetic arousal and calmness are representative of the person’s average as they were answered once to multiple times per day and the different parameters of affect are known to change throughout the day (Reichert et al., 2020). Finally, the selection of epoch lengths is important to consider for PA estimations by accelerometers, especially if both adults and children are included in the study (Fiedler et al., 2021). The choice of another epoch length (e.g., 10 s epochs instead of 60 s epochs) might have led to differing findings for intensity-related parameters (i.e., MVPA).

## Conclusion

The study expands previous findings from studies examining the dynamic relations of physical activity (PA), sleep quality and affective states by considering the whole day instead of shorter timeframes, focusing on multiple outcome parameters and predictors during an intervention period, and by including both adults and children as participants of the study. The results confirm that every one unit increase in self-rated energetic arousal and valence was associated with 336.23 and 489.64 higher step count per day respectively, while every one unit increase in valence was associated with 6.55 more minutes of moderate to vigorous PA (MVPA), while energetic arousal was not associated with MVPA. In addition, a one unit increase in calmness was associated with 244.11 fewer steps and 2.07 fewer minutes MVPA per day. The additional exploration found sex, age, non-wear time, and the differentiation between weekday and weekend as important covariates and control variables for PA. Overall, this study shows that affective states are important predictors for PA and should be included in the development of effective mHealth interventions to facilitate health behavior change. Future EMA studies should explore the dose–response relationship for predictors and covariates of PA, while future intervention studies should consider the known associations between predictors and PA as possible targets for individual tailoring of the interventions. In doing so, barriers for PA uptake can be identified and targeted by including the individual needs for each person under a variety of circumstances into the equation and form the basis for highly individualized just-in-time adaptive interventions.

## Supplementary Information

Supplement figures 1 to 7 display an overview of daily measurement variance for all included outcomes and predictors (Fig. 1), and the individual development of those values for each participant seperated by outcome/predictor (Fig. 2–7).

## Declarations

All procedures performed in studies involving human participants or on human tissue were in accordance with the ethical standards of the institutional and/or national research committee (Institutional Review Board (or Ethics Committee) of the Karlsruhe Institute of Technology (28 November 2019)) and with the 1975 Helsinki declaration and its later amendments or comparable ethical standards. Informed consent was obtained from all individual participants (children and legal guardians) included in the study.
