# Supplementary material for: Sleep quality, valence, energetic arousal, and calmness as predictors of device-based measured physical activity during a three-week mHealth intervention: An ecological momentary assessment study within the SMARTFAMILY trial
Source: Ger J Exerc Sport Res. 2022 Apr 14;52(2):237–47. doi: 10.1007/s12662-022-00809-y (PMC9008661; doi:10.1007/s12662-022-00809-y)
Supplement: Supplementary file 1 — Supplement figures 1 to 7 display an overview of daily measurement variance for all included outcomes and predictors (Fig. 1), and the individual development of those values for each participant seperated by outcome/predictor (Fig. 2–7). [file 12662_2022_809_MOESM1_ESM.docx]

**Sleep quality, valence, energetic arousal, and calmness as predictors of device-based measured physical activity during a three-week mHealth intervention: An ecological momentary assessment study within the SMART*FAMILY* trial.**

Janis Fiedler*^1^, Caroline Seiferth^2^, Tobias Eckert^1,3^, Alexander Woll^1^ and Kathrin Wunsch^1^

^1^Institute of Sport and Sport Science, Karlsruhe Institute of Technology, 76131 Karlsruhe, Germany;

^2^Department of Clinical Psychology and Psychotherapy, University of Bamberg, 96047 Bamberg, Germany

^3^Heidelberg Institute of Global Health (HIGH), Heidelberg University, 69117 Heidelberg, Germany

caroline.seiferth@uni-bamberg.de; tobias.eckert@uni-heidelberg.de; alexander.woll@kit.edu; kathrin.wunsch@kit.edu

*Correspondence: janis.fiedler@kit.edu; Tel.: +49-721-608-46978; https://orcid.org/0000-0002-9291-2191

German Journal of Exercise and Sport Research

**
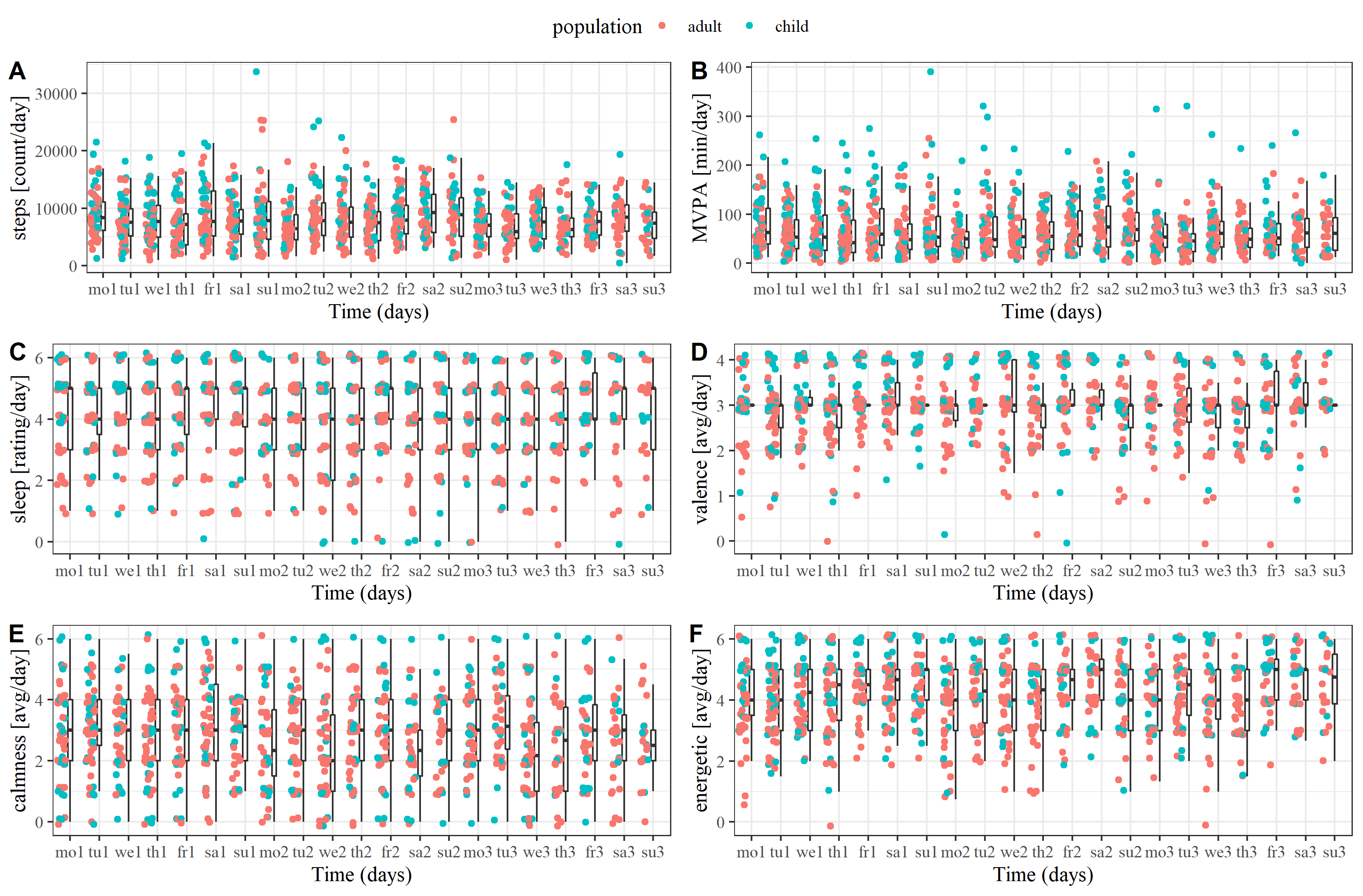
Figure 1.** *Descriptive daily person mean values (slightly jittered for better visualization) of A: daily average step count (steps), B: daily average minutes moderate to vigorous physical activity (MVPA), C: daily sleep quality (sleep), D: daily average (avg) valence (valence), E: daily average calmness (calmness), and F: daily average energetic arousal (energetic) during the three-week intervention period (mo = Monday, tu = Tuesday, we = Wednesday, th = Thursday, fr = Friday, sa = Saturday, su = Sunday, 1 = week one, 2 = week two, 3 = week three) for children (blue) and adults (red) of the SMARTFAMILY study.*

*
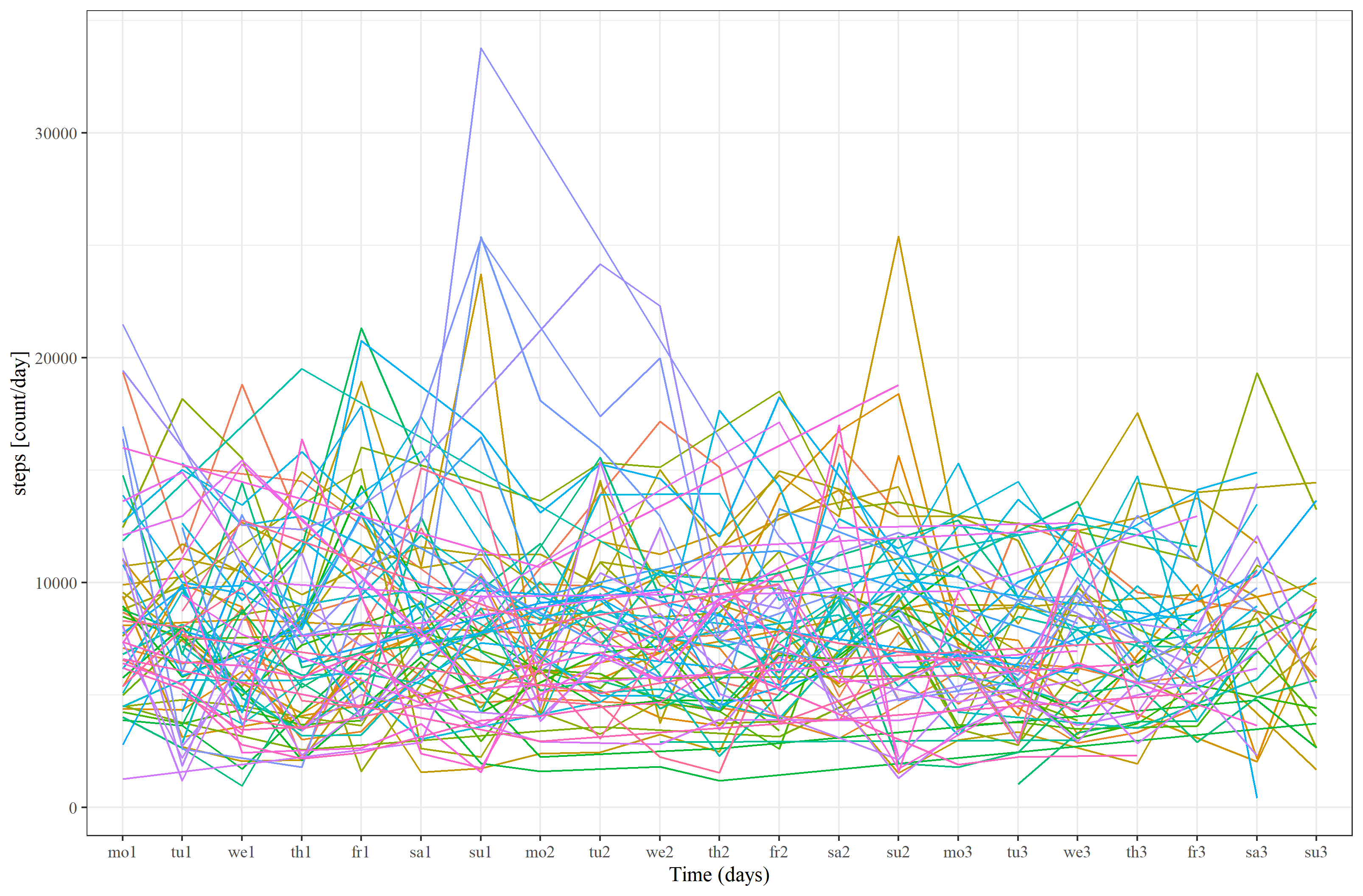
***Figure 2.** *Descriptive values of daily average step count (steps) for each participant (each line represents one participant) during the three-week intervention period (mo = Monday, tu = Tuesday, we = Wednesday, th = Thursday, fr = Friday, sa = Saturday, su = Sunday, 1 = week one, 2 = week two, 3 = week three) of the SMARTFAMILY study.*

*
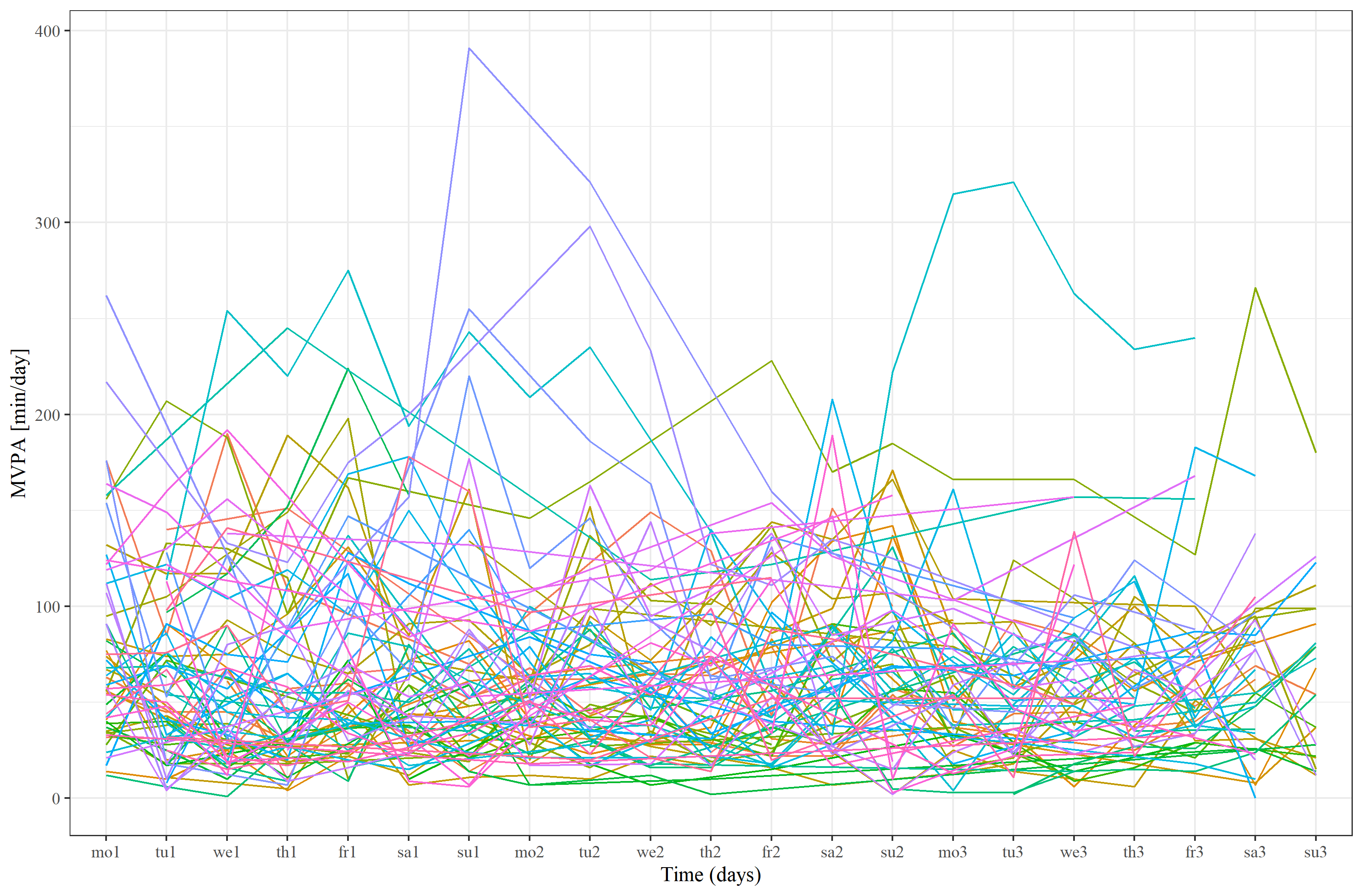
*

**Figure 3.** *Descriptive values of daily average minutes of moderate-to-vigorous physical activity (MVPA) for each participant (each line represents one participant) during the three-week intervention period (mo = Monday, tu = Tuesday, we = Wednesday, th = Thursday, fr = Friday, sa = Saturday, su = Sunday, 1 = week one, 2 = week two, 3 = week three) of the SMARTFAMILY study.*


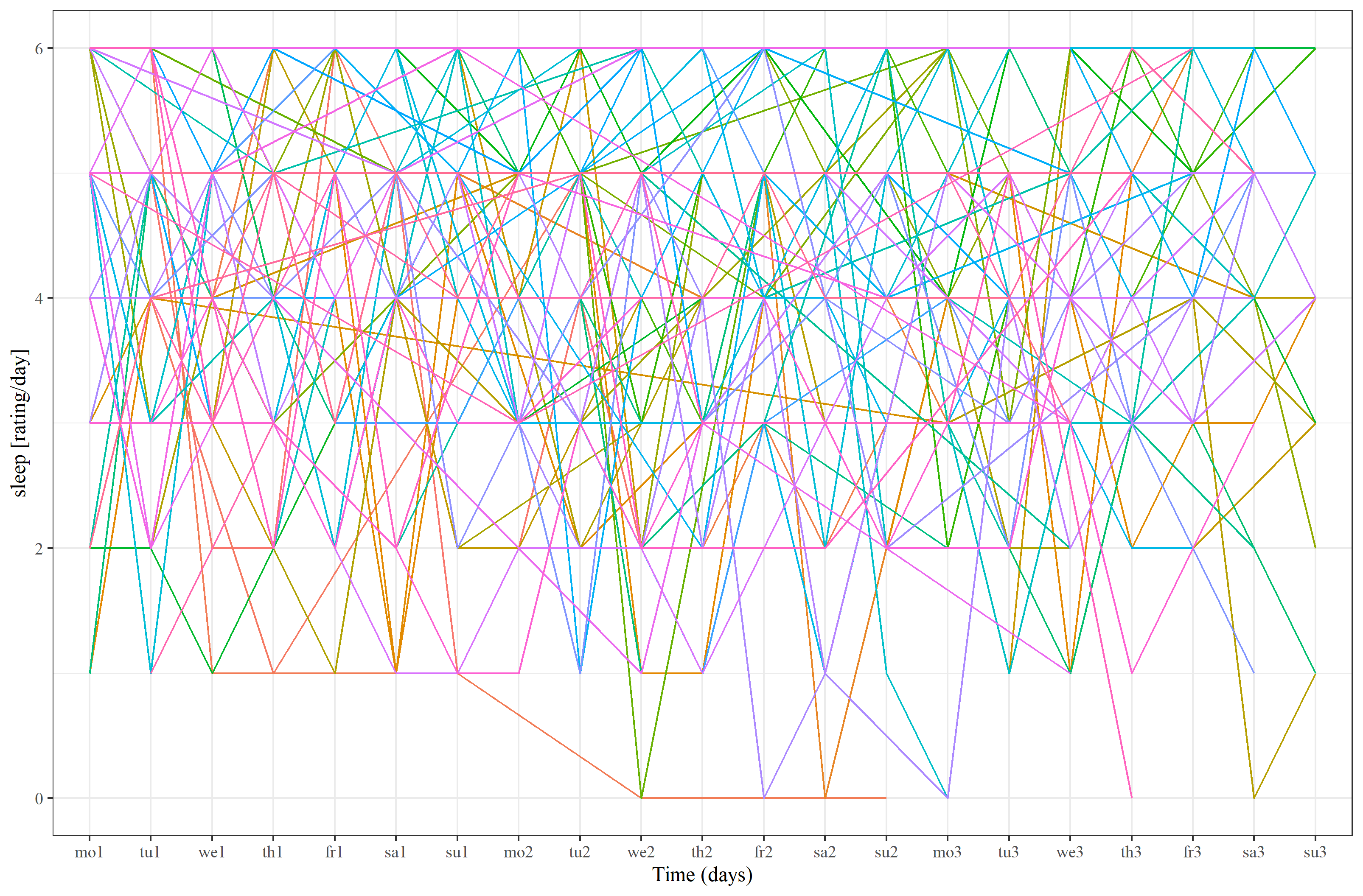
**Figure 4.** *Descriptive values of daily sleep quality (sleep) ratings for each participant (each line represents one participant) during the three-week intervention period (mo = Monday, tu = Tuesday, we = Wednesday, th = Thursday, fr = Friday, sa = Saturday, su = Sunday, 1 = week one, 2 = week two, 3 = week three) of the SMARTFAMILY study.*

*
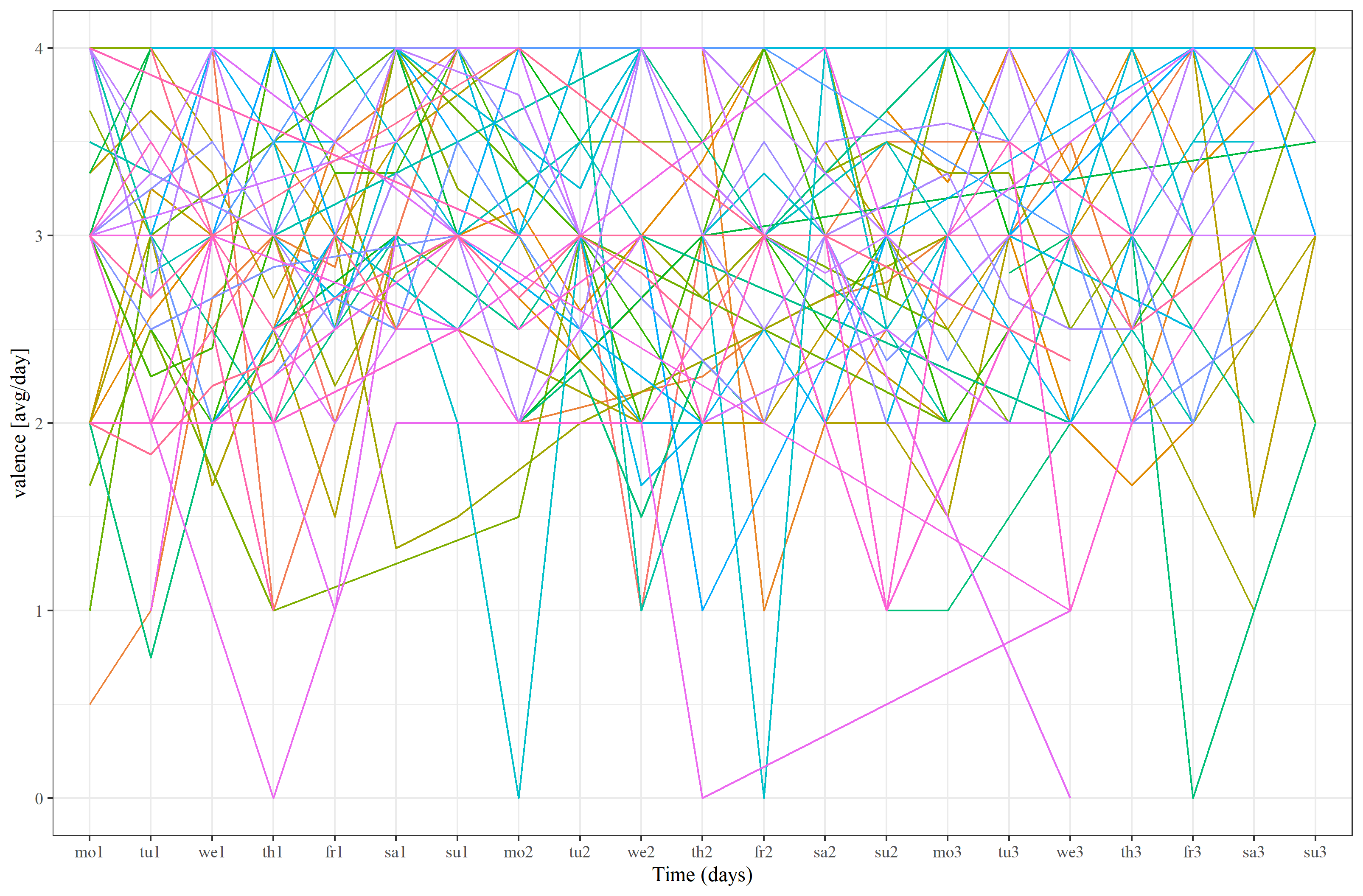
*

**Figure 5.** *Descriptive values of daily average valence (valence) for each participant (each line represents one participant) during the three-week intervention period (mo = Monday, tu = Tuesday, we = Wednesday, th = Thursday, fr = Friday, sa = Saturday, su = Sunday, 1 = week one, 2 = week two, 3 = week three) of the SMARTFAMILY study.*


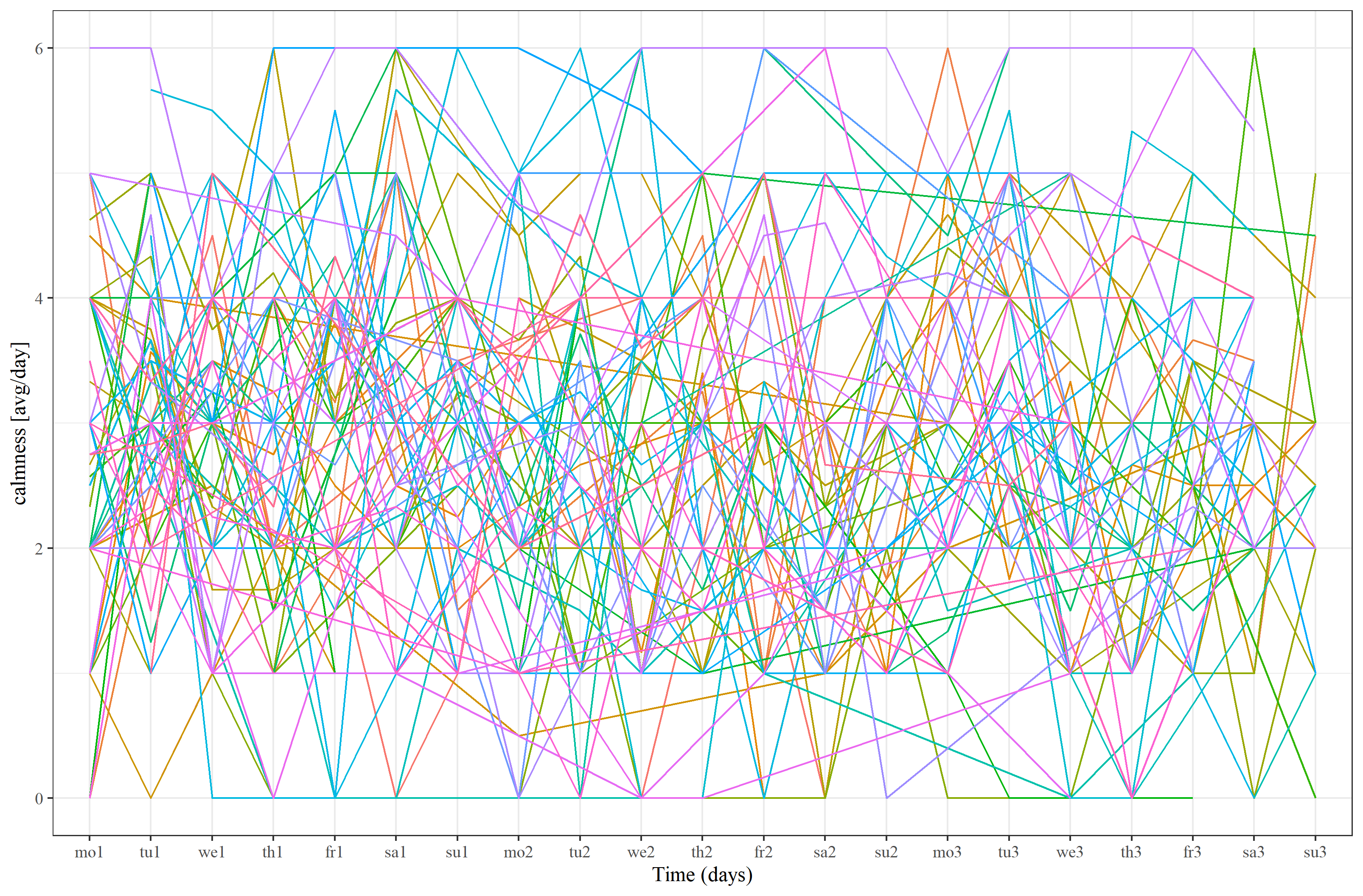


**Figure 6.** *Descriptive values of daily average calmness (calmness) for each participant (each line represents one participant) during the three-week intervention period (mo = Monday, tu = Tuesday, we = Wednesday, th = Thursday, fr = Friday, sa = Saturday, su = Sunday, 1 = week one, 2 = week two, 3 = week three) of the SMARTFAMILY study.
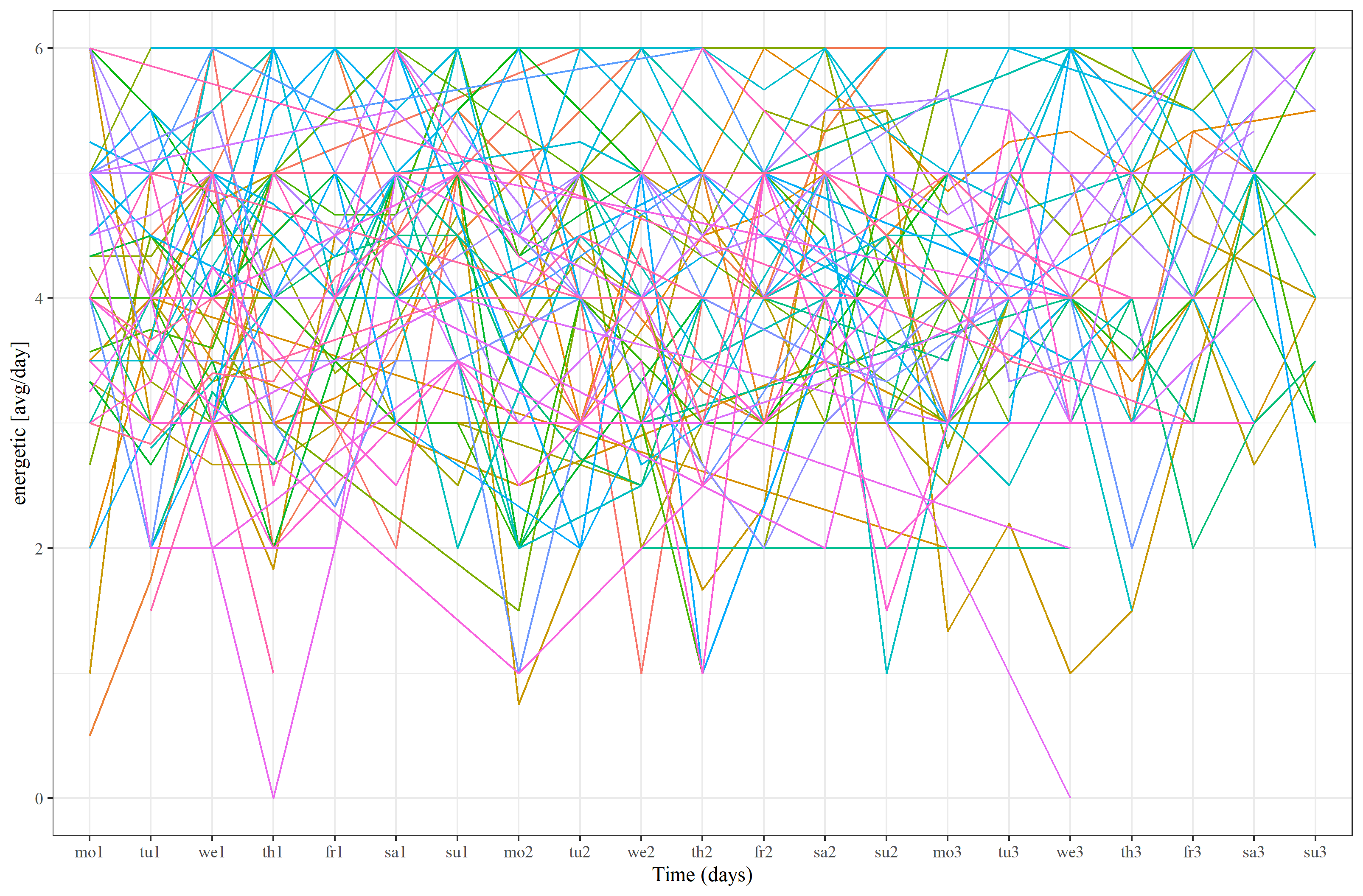
***Figure 7.** *Descriptive values of daily average energetic arousal (energetic) for each participant (each line represents one participant) during the three-week intervention period (mo = Monday, tu = Tuesday, we = Wednesday, th = Thursday, fr = Friday, sa = Saturday, su = Sunday, 1 = week one, 2 = week two, 3 = week three) of the SMARTFAMILY study.*
